# Enhanced annotation of CD45RA to distinguish T cell subsets in single-cell RNA-seq via machine learning

**DOI:** 10.1093/bioadv/vbad159

**Published:** 2023-11-06

**Authors:** Ran Ran, Douglas K Brubaker

**Affiliations:** Department of Pathology, Center for Global Health and Diseases, Case Western Reserve University School of Medicine, Cleveland, OH 44106, United States; Department of Pathology, Center for Global Health and Diseases, Case Western Reserve University School of Medicine, Cleveland, OH 44106, United States; The Blood, Heart, Lung, and Immunology Research Center, Case Western Reserve University, University Hospitals of Cleveland, Cleveland, OH 44106, United States

## Abstract

**Motivation:**

T cell heterogeneity presents a challenge for accurate cell identification, understanding their inherent plasticity, and characterizing their critical role in adaptive immunity. Immunologists have traditionally employed techniques such as flow cytometry to identify T cell subtypes based on a well-established set of surface protein markers. With the advent of single-cell RNA sequencing (scRNA-seq), researchers can now investigate the gene expression profiles of these surface proteins at the single-cell level. The insights gleaned from these profiles offer valuable clues and a deeper understanding of cell identity. However, CD45RA, the isoform of CD45 which distinguishes between naive/central memory T cells and effector memory/effector memory cells re-expressing CD45RA T cells, cannot be well profiled by scRNA-seq due to the difficulty in mapping short reads to genes.

**Results:**

In order to facilitate cell-type annotation in T cell scRNA-seq analysis, we employed machine learning and trained a ${\text{CD}45\text{RA}}^{+/-}$ classifier on single-cell mRNA count data annotated with known CD45RA antibody levels provided by cellular indexing of transcriptomes and epitopes sequencing data. Among all the algorithms we tested, the trained support vector machine with a radial basis function kernel with optimized hyperparameters achieved a 99.96% accuracy on an unseen dataset. The multilayer perceptron classifier, the second most predictive method overall, also achieved a decent accuracy of 99.74%. Our simple yet robust machine learning approach provides a valid inference on the CD45RA level, assisting the cell identity annotation and further exploring the heterogeneity within human T cells. Based on the overall performance, we chose the support vector machine with a radial basis function kernel as the model implemented in our Python package scCD45RA.

**Availability and implementation:**

The resultant package scCD45RA can be found at https://github.com/BrubakerLab/ScCD45RA and can be installed from the Python Package Index (PyPI) using the command “pip install sccd45ra.”

## 1 Introduction

T cells play a pivotal role in adaptive immunity, serving as the cornerstone of the body’s defense mechanism against pathogens (Denman 1992). Each T cell clone has unique, sophisticatedly rearranged T cell receptors (TCRs) expressed on their surface, allowing them to bind to a specific group of antigens sourced from abnormal cells or foreign organisms and initiate an immune response (Alcover *et al.* 2018). Also, T cells are a remarkably heterogeneous population that can be further divided into subsets, such as CD4/CD8 T cells if by their surface glycoproteins, αβ/*γδ* T cell by their TCR chains, or naive/stem/memory/effector by their functions (Beverley 1987, Fowell *et al.* 1991, Allison 1993, Kemeny *et al.* 1999). Those subsets play different roles in maintaining immune system homeostasis and orchestrating immune response. Such heterogeneity underscores their versatility but also poses significant challenges to accurate cell characterization, which is important for developing strategies for ameliorating or reversing diseases.

Historically, immunologists have relied on protein-targeted techniques such as immunofluorescence, western blot, and flow cytometry to distinguish T cell subtypes in blood/peripheral tissue samples (Denman 1992). Those tools enable the characterization of cells based on a well-established set of surface protein markers, including but not limited to CD4, CD8, CD25, CD45, CD127, CCR7, CD62L, CD45RO, and CD45RA (Beverley 1987, Perfetto *et al.* 2004). By analyzing the expression patterns of these markers, scientists have been able to classify T cells into helper T cells, cytotoxic T cells, regulatory T cells, memory T cells, etc. (Zhu *et al.* 2010, Wherry and Kurachi 2015), and each is proved to have distinct functions and roles within the immune system. The development of techniques like single-cell RNA sequencing (scRNA-seq) has enabled the discovery of novel T cells subpopulations at an even higher resolution and provided a deeper understanding of their distinct functions in the immune system (Papalexi and Satija 2018). Specifically, scRNA-seq reports the counts of captured mRNA as the indicator of gene’s transcriptional activities in every single cell in the sample (Zheng *et al.* 2017). Under the assumption that the same type of cells has a similar pattern of gene expression, unsupervised clustering on the feature matrix generates clusters that are the union of cells of presumably the same cell type, which provides insights into cell subtypes identification (Kiselev *et al.* 2017).

Despite these advances, certain limitations persist in the application of the scRNA-seq for T cells studies. One such challenge is the accurate profiling of CD45RA, an isoform of the CD45 protein. CD45RA is the result of alternative splicing of CD45 and serves as a canonical marker to distinguish between naive T/central memory T (TCM) cells (naive T is ${\text{CD}45\text{RA}}^{+}$ while TCM is ${\text{CD}45\text{RA}}^{-}$) and effector memory T (TEM)/effector memory re-expressing CD45RA T (TEMRA) cells (TEMRA is ${\text{CD}45\text{RA}}^{+}$ while TEM is ${\text{CD}45\text{RA}}^{-}$) (Traag *et al.* 2019). However, the expression of CD45RA mRNA can be challenging to report using scRNA-seq techniques because most of them rely on next-generation sequencing (NGS) platforms that fragment the cDNA of mRNA and obtain gene information by mapping the short reads to the human genome in the downstream analysis (Goodwin *et al.* 2016, Hardwick *et al.* 2019). Therefore, it is not uncommon for CD45RA information to be missing or less reliable in the output count matrix from scRNA-seq, which adds difficulties to the optimal annotation of T cell subsets identities. To overcome this limitation, researchers have turned to alternative approaches, including the use of multiomic techniques that combine scRNA-seq with other technologies, such as cellular indexing of transcriptomes and epitopes by sequencing (CITE-seq) (Stoeckius *et al.* 2017). CITE-seq enables simultaneous measurement of surface protein expression and gene expression at the single-cell level, offering a more comprehensive view of cellular identity. Instead of reporting the level of CD45RA mRNA, CITE-seq can measure the product, alias the CD45RA protein, along with the RNA profiles.

Still, a method to better infer the CD45RA level in conventional scRNA-seq experiments is needed for studying human immunology. Retrospectively, there is likely important information about CD45RA identified in studies performed prior to the wide implementation of CITE-seq, where such information would re-contextualize and enhance the interpretation of previously collected data. Prospectively, even as CITE-seq gains popularity, there will likely be biological use cases where the technique is cost prohibitive. Such methods for inferring CD45RA status are potentially important for immunology in the future and may need to be expanded to other markers besides CD45RA. Given the production of protein is the result of a series of highly orchestrated gene expressions, an intuitive hypothesis is that ${\text{CD}45\text{RA}}^{+}$ cells should have a different pattern in mRNA counts compared to ${\text{CD}45\text{RA}}^{-}$ cells. Therefore, we employed machine learning approaches and trained multiple ${\text{CD}45\text{RA}}^{+/-}$ classifiers on the NGS single-cell mRNA count matrix with known CD45RA antibody levels reported by CITE-seq to facilitate cell type annotation in T cell NGS scRNA-seq analysis.

## 2 Methods

### 2.1 Data acquisition

Raw CITE-seq counts of 5559 healthy adult peripheral blood mononuclear cells labeled with CD45RA antibody level were obtained from NCBI Gene Expression Omnibus series GSE144434 as the training/testing dataset (Pont *et al.* 2020). To test the performance and robustness of models on NGS scRNA-seq data generated by different experiments, the cytometry by time of flight-sorted scRNA-seq raw counts of ${\text{CD}45\text{RA}}^{-}$ T cells in healthy human blood from study GSE150132 (Rasouli *et al.* 2020) and the PBMC CITE-seq data from study GSE164378 (Hao *et al.* 2021) were obtained.

### 2.2 Single-cell RNA-seq data processing

The downstream analysis was done with scanpy (v1.9.1) (Wolf *et al.* 2018) on Python and Seurat (v4.2.0) on R (Stuart *et al.* 2019). GSE164378 was down-sampled from 160k cells to 80k cells by random selection to be more computation-effective. Cells with CD19 or without CD3D/E/G/Z expression were discarded only to keep the profiles of T cells. GSE150132 and GSE164378 were already preprocessed (quality control) by the data provider, so the preprocessing steps below are for the GSE144434 dataset on which models were trained. Low-quality cells were already dropped by the data provider based on their extremely low UMI counts (min counts = 1000), high mitochondrial gene counts (mt-genes fraction > 20%), and low number of uniquely expressed genes (min genes = 200). In this study, cells with less than 5% of the ribosomal gene count were also dropped. Genes expressed in less than two cells in each dataset were excluded. Multiplets were removed by Scrublet (v0.2.3) (Wolock *et al.* 2019).

A cell cycle score was calculated for each cell in the preprocessed three datasets based on the expression of cell cycle-related genes as described previously (Macosko *et al.* 2015). All datasets were normalized by SCTransform (v0.3.5) on R (Hafemeister and Satija 2019). Cell mitochondrial fraction and the difference between the S phase score and the G2M phase score, as proposed as the representation of the cell cycle score, were regressed out during the normalization of datasets. Three thousand highly variable features were calculated and used to perform the principal component analysis (PCA, 50 pcs) for each dataset. A neighborhood graph of observations was computed on the 50 PCs of each dataset with the size of local neighbors set to 15. Leiden clustering (resolution = 0.5) was performed based on the computed neighborhood graph of observations to reveal the general subtypes of the T cells (McInnes *et al.* 2018, Traag *et al.* 2019). Partition-based graph abstraction based on the Leiden clusters, with a threshold of 0.2, was used to initialize every uniform manifold approximation (UMAP, 50 pcs, min_dist = 0.01, spread = 2, n_components = 2, alpha = 1.0, gamma = 1.0) to facilitate the convergence of manifold (Wolf *et al.* 2019).

### 2.3 Determine CD45RA positive/negative label

The CD45RA level reported by CITE-seq is derived from the counts of unique DNA-barcoded sequences associated with CD45RA antibodies (Stoeckius *et al.* 2017). Unspecified parameters of the functions used in this study were kept at their default values. It has been preprocessed, normalized, and log-transformed by the data provider. The Otsu method, also known as Otsu’s thresholding, in the skimage library (Walt *et al.* 2014) is used to calculate the optimum threshold $t^{*}$ of CD45RA level to separate positive and negative cells. Specifically, the Otsu method aims to find a threshold $t^{*}$ that maximizes the between-class variance while implicitly minimizing the within-class variance. Since the overlapping observations (i.e. cells that had a CD45RA level close to $t^{*}$) were hard to be assigned a positive/negative label, to reduce the false positivity/negativity in ${\text{CD}45\text{RA}}^{+/-}$ labeling, ${\text{CD}45\text{RA}}^{+}$ cells were defined as cells having a CD45RA level ⩾ $t^{*}+0.5$, and ${\text{CD}45\text{RA}}^{-}$ cells were defined as cells having a CD45RA level ⩽ $t^{*}-0.5$. In other words, overlapping observations were not used in the model training.

### 2.4 Train-test data split

The normalized and scaled GSE144434 CITE-seq data was split into training and testing sets with a 0.2 test size and a random state of 42 using scikit-learn (v1.1.2) in Python (Fabianpedregosa *et al.* 2011). Unless other specified, data were split in such a way during the model training and cross-validation.

### 2.5 Differentially expressed genes identification

One of our main hypotheses is that the gene expression pattern of CD45RA positive and negative cells should differ. Differentially expressed gene (DEG) analysis is an established computational methodology that has been widely employed in different studies for the rigorous statistical identification of genes that exhibit significant variations in expression levels across distinct experimental conditions? DEG analysis in Monocle 3 Mon involves fitting a generalized linear model (GLM) to the SCTransform-corrected counts (Trapnell *et al.* 2022). The quasi-Poisson distribution is used as the noise model to account for both the mean and variance in the data.

For a given gene, let *y_ij_* represent the observed expression level for cell *i* in condition *j*, where i=1,2,…,n, and *j *=* *1, 2 (two conditions, ${\text{CD}45\text{RA}}^{+/-}$). The quasi-Poisson GLM can be written as


(1) $$y_{ij}∼\text{QuasiPoisson}(\mu_{ij},\phi )$$

(2) $$\text{log}(\mu_{ij})=X_{ij}\beta_{j},$$

where *μ_ij_* is the expected expression level for cell *i* in condition *j*, ϕ is the dispersion parameter, *X_ij_* is the design matrix representing covariates (e.g. experimental conditions, batch effects), and $\beta_{j}$ is the vector of regression coefficients for condition *j*.

The link function is the natural logarithm, which maps the expected expression level (*μ_ij_*) to the linear predictor ($X_{ij}\beta_{j}$), helping ensure that the expected expression level is always nonnegative.

The dispersion parameter (ϕ) accounts for the overdispersion in the data, which occurs when the variance is greater than the mean. In the quasi-Poisson model, the variance is modeled as


(3) $$\text{Var}(y_{ij})=\mu_{ij}+\phi \mu_{ij}^{2}.$$

For each gene, the GLM is fit to the data using maximum likelihood estimation, which involves finding the $\beta_{j}$ and ϕ values that maximize the likelihood of the observed data.

A likelihood ratio test is performed to test for differential expression between the two conditions. This compares the likelihood of the data under the full model (with separate $\beta_{j}$ values for each condition) to the likelihood under the null model (with the same $\beta_{j}$ value for both conditions). The test statistic is calculated as follows:


(4) $$D=2\times (\text{log}(L_{\text{full}})-\text{log}(L_{\text{null}})),$$

where *D* follows a chi-square distribution with degrees of freedom equal to the difference in the number of parameters between the full and null models. A *q*-value is calculated from the test statistic, and genes with *q*-values below 0.05 are considered DE genes.

### 2.6 Feature selection

After splitting data into training and testing sets, in the training set, features were selected from two sources. The first source was the DE genes between ${\text{CD}45\text{RA}}^{+/-}$ groups. The fold change of a DE gene’s level in one group with respect to the other could be quantified to describe the extent of differential expression. DE genes with a fold change above an arbitrary threshold, $t_{\text{DE}}$, were selected as input features for the classifier. By adjusting $t_{\text{DE}}$, it became possible to fine-tune the input features’ degree of distinction across ${\text{CD}45\text{RA}}^{+/-}$.

Mathematically, given the SCTransform-corrected gene expression values in ${\text{CD}45\text{RA}}^{+}$ (*C*_1_) and ${\text{CD}45\text{RA}}^{-}$ (*C*_2_), the fold change (FC) for a specific gene can be calculated as follows:


(5) $$\text{FC}=\frac{\text{Average expression level in}\;C_{2}}{\text{Average expression level in}\;C_{1}}.$$

The log2-transformed fold change is defined as


(6) $${\;\text{log}\;}_{2}\text{FC}={\;\text{log}\;}_{2}\frac{\text{Average expression level in}\;C_{2}}{\text{Average expression level in}\;C_{1}}.$$

In the context of the quasi-Poisson GLM, the log_2_ fold change can be estimated by calculating the difference in the estimated coefficients, $\beta_{j}$, between the two conditions:


(7) $${\;\text{log}\;}_{2}FC=\beta_{C_{2}}-\beta_{C_{1}}.$$

The second source for feature selection comes from previous biological knowledge. Heterogeneous nuclear ribonucleoprotein L-like (hnRNPLL) is an RNA-binding protein that plays a crucial role in alternative splicing, a process where premessenger RNA (pre-mRNA) is rearranged to produce different mRNA molecules and, consequently, various protein isoforms. hnRNPLL has been implicated in the regulation of CD45 alternative splicing, specifically in the generation of the CD45RA isoform. hnRNPLL binds to specific RNA sequences in the CD45 pre-mRNA, promoting the inclusion or exclusion of specific exons. The binding of hnRNPLL to CD45 pre-mRNA has been shown to enhance the exclusion of variable exons, leading to the generation of the CD45RA isoform (Oberdoerffer *et al.* 2008). In this manner, hnRNPLL helps regulate the expression of CD45 isoforms and contributes to the proper functioning of the immune system.

Therefore, we hypothesized that hnRNPLL and genes correlated with it may be indicative of the CD45RA status. Spearman correlation coefficients of every gene’s normalized and scaled expression with hnRNPLL’s expression were calculated. Genes that had an absolute correlation coefficient with hnRNPLL in the $P_{\text{hn}}$ percentile with a *P*-value < 0.05 were selected as input features. Similar to the DE genes selection, the extent of the input features correlate with hnRNPLL can also be optimized later by adjusting $P_{\text{hn}}$.

### 2.7 Classifier training

#### 2.7.1 Support vector machine

Support vector machine (SVM) is a supervised learning method often used for classification and regression problems (Vapnik 2000). It aims to find the optimal hyperplane to classify the data points while maximizing the margin, the closest distance from data points from each class to the hyperplane. SVM can efficiently process sparse data in high-dimensional spaces by relying on the inner product between feature vectors. Such a technique known as the “kernel trick” allows SVM to implicitly map data into higher-dimensional feature spaces for more effective linear decision boundaries. Furthermore, maximal margin separation enables SVM to find the optimal hyperplane in high-dimensional spaces, which helps generalize well to unseen data. Lastly, SVM incorporates regularization to prevent overfitting.

Training data were fitted to an SVM with a radial basis function (RBF) kernel and another SVM with a linear kernel using scikit-learn (v1.1.2) in Python (Fabianpedregosa *et al.* 2011). For the SVM with RBF kernel, $t_{\text{DE}}$ and $P_{\text{hn}}$ that threshold the input features, along with the cost $C_{\text{SVM}}$ and the RBF kernel coefficient *γ* were optimized by a Bayesian optimizer using Bayesian optimization in Python Nog (Nogueira 2014). The Bayesian optimizer used a Gaussian process as the surrogate model with a Matérn kernel. Priors were not applied to the hyperparameters. The optimizer iterated for a maximum of 30 times or until the expected improvement was below a threshold of ${1e}^{-6}$. We chose 5-fold stratified cross-validation to ensure balanced classes across folds. The average accuracy across the folds was the primary metric used to evaluate and select the hyperparameters. After determining the optimal hyperparameters, the final SVM model was retrained on the entire training dataset. Unless otherwise specified, the Bayesian optimizer used anywhere in this study follows the same rule. $t_{\text{DE}}\in (1,4)$ or $\left(-4,-1),\;P_{\text{hn}}\in (1e-6,0.1),C_{\text{SVM}}\in (1e-6,100),\;\gamma \in (1e-6,2\right)$. For the linear kernel SVM, no kernel coefficient was needed.

Eventually, the final SVM was trained on the feature-optimized training set and took the optimized hyperparameters. It was then used to predict the CD45RA label of testing data, unseen ${\text{CD}45\text{RA}}^{-}$ data. The accuracy of the prediction on datasets with known CD45RA labels was reported, and the performance on classifying single-cell data of two SVMs were compared.

#### 2.7.2 Logistic regression

Logistic regression (LR) is a classic and relatively simple binary classifier (Cox 1958). It can handle high-dimensional data since it relies on finding the optimal decision boundary, which is not affected by the “curse of dimensionality” in the same way as distance-based measures (King and Zeng 2001). Also, LR is adept at handling the sparse scRNA-seq counts due to its ability to incorporate regularization, which encourages sparse solutions and helps in feature selection.

The training data were fitted to an LR classifier with a threshold for the predicted probabilities of 0.5 using scikit-learn (v1.1.2) in Python (Fabianpedregosa *et al.* 2011). $t_{\text{DE}}$ and $P_{\text{hn}}$ that threshold the input features, along with the regularization strength *C* were optimized by a Bayesian optimizer using Bayesian optimization in Python Nog, aiming to achieve the optimal 5-folds cross-validation averaged training accuracy in 30 iterations (Nogueira 2014). $t_{\text{DE}}\in (1,4)$ or $\left(-4,-1),\;P_{\text{hn}}\in (1e-6,0.1),\;C_{\text{LR}}\in (1e-6,2\right)$.

Eventually, the final LR classifier was trained on the feature-optimized training set and took the optimized hyperparameters. It was then used to predict the CD45RA label of testing data, unseen ${\text{CD}45\text{RA}}^{-}$ data. The accuracy of the prediction on datasets with known CD45RA labels was reported.

#### 2.7.3 Support vector machine stacked logistic regression

Stacking an LR model and an SVM together can potentially create a more powerful classifier by leveraging the strengths of both models (Wolpert 1992). LR is a linear model that works well when the decision boundary between classes is relatively linear. At the same time, SVM, particularly with nonlinear kernels like RBF, can capture more complex decision boundaries.

We trained a meta LR using the predictions of the LR and SVM classifiers as input features and the true ${\text{CD}45\text{RA}}^{+/-}$ labels as the target variable using scikit-learn (v1.1.2) in Python (Fabianpedregosa *et al.* 2011). $t_{\text{DE}}$ and $P_{\text{hn}}$ that threshold the input features, along with the regularization strength $C_{\text{LR}}$ from LR, the cost $C_{\text{SVM}}$ and the RBF kernel coefficient *γ* from SVM were optimized by a Bayesian optimizer using Bayesian optimization in Python Nog, aiming to achieve the optimal 5-folds cross-validation averaged training accuracy in 30 iterations. $t_{\text{DE}}\in (1,4)$ or $\left(-4,-1),\;P_{\text{hn}}\in (1e-6,0.1),\;C_{\text{SVM}}\in (1e-6,100),\;\gamma \in (1e-6,2),C_{\text{LR}}\in (1e-6,2\right)$. Since the meta LR only had two features, which were the predictions by SVM and LR, its hyperparameter was not optimized.

The final model was trained on the feature-optimized training set and took the optimized hyperparameters. It was then used to predict the CD45RA label of testing data, unseen ${\text{CD}45\text{RA}}^{-}$ data. The accuracy of the prediction on datasets with known CD45RA labels was reported.

#### 2.7.4 Multilayer perceptron

The multilayer perceptron (MLP) is an artificial neural network characterized by its multiple layers of interconnected neurons (Rumelhart *et al.* 1986). This machine learning model has proven its mettle in various applications, including binary classification tasks (Park and Kanehisa 2003, Moreno *et al.* 2011, Ali and Al-Hroot 2016). In comparison to LR, the MLP excels in its ability to model nonlinear relationships. While LR is effective in linear contexts, its simplicity restricts its capabilities in handling more complex data. The MLP, on the other hand, can learn nonlinear patterns with ease, providing excellent performance in cases where linearity cannot be assumed. When it comes to SVM, while SVM can handle nonlinear data through the use of kernels, it lacks the flexibility and scalability inherent to the MLP’s architecture. The MLP can be fine-tuned to a wide range of classification problems by adjusting the number of hidden layers and neurons. This adaptability makes the MLP more robust for tackling diverse and challenging datasets.

For simplicity, we used the optimized features in linear and RBF kernel SVM as the input features for two MLPs, respectively. The training data were fitted to an MLP threshold for the predicted probabilities of 0.5 using tensorflow (v2.12.0) in Python (Abadi *et al.* 2016). ReLu was used as the activation function for all layers except the output layer, which used sigmoid as its activation function. The epoch number was 15, and the batch size was 32 when building the MLP. The number of layers, the number of neurons, the learning rate, and the dropout rate were optimized by a Bayesian optimizer with the aim of achieving minimal average binary cross-entropy of the 5-fold cross-validation in 30 iterations. $N_{\text{layers}}\in (1,3),\;N_{\text{nodes}}\in (16,128),\;\eta \in (1e-4,0.01),\rho \in (0.1,0.5)$. The final MLP was trained on the feature-optimized training set and took the optimized hyperparameters. It was then used to predict the CD45RA label of testing data, unseen ${\text{CD}45\text{RA}}^{-}$ data. The accuracy of the prediction on datasets with known CD45RA labels was reported.

#### 2.7.5 scCD45RA running time benchmarking

An average-performance computer [Intel(R) Core(TM) i5-8300H CPU @ 2.30 GHz, 16 Gb RAM] was used to perform the test. Running time was measured for five individual running. For every round of running, the kernel was restarted and data were reloaded. The running time was the difference of Python “time” time stamp difference before and after the command “cd45ra_infer.”

#### 2.7.6 Seurat 4 multimodal reference mapping

Seurat v4 was employed for the reference mapping of scRNA-seq datasets to annotated references. Normalized and log-transformed validation set and GSE150132 data were mapped to a CITE-seq reference that had been characterized by 228 antibodies across 162 000 PBMCs. Anchors between the reference and query were identified using the FindTransferAnchors() function, where a supervised PCA (spca) transformation was employed. Lastly, cell type labels and protein data were transferred from the reference to the query, and the query data was projected onto the UMAP structure of the reference using the MapQuery() function.

## 3 Results

### 3.1 Features interpretation

**Figure 1.**
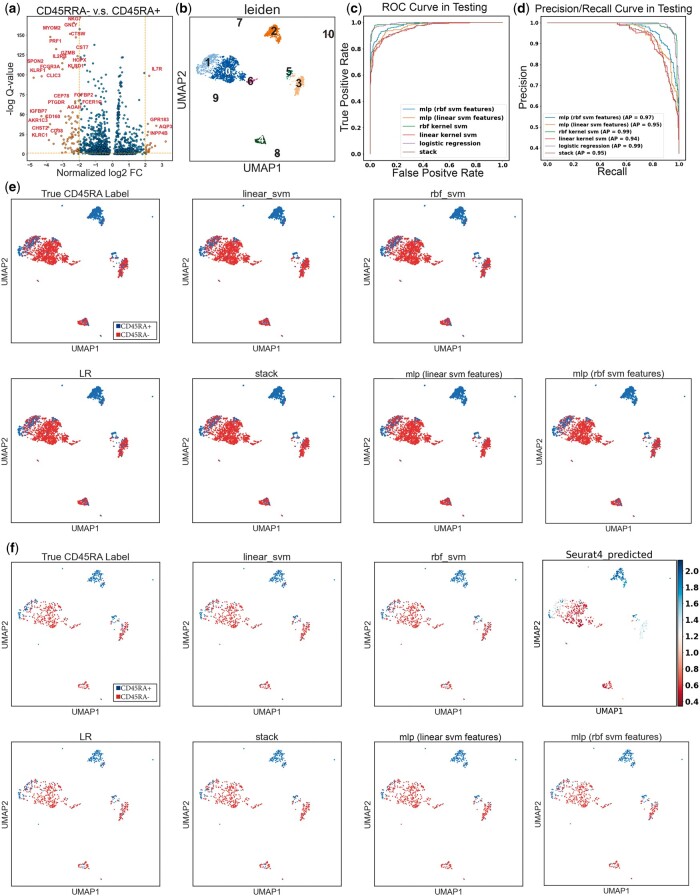
Overview of the features, the data, and the prediction made by classifiers. (a) Volcano plot of the DEGs. *x*-axis: gene expression level log_2_ fold change (log_2_FC) in CD45RA${}^{-}$ cells with respect to CD45RA${}^{+}$ cells. *y*-axis: −log_10_*q*-value (false discovery rate) of gene’s fold change in CD45RA${}^{-}$ with respect to CD45RA${}^{+}$. The threshold of the magnitude of the DE genes’ log_2_FC across CD45RA${}^{+/-}$ to be considered distinguished enough to be reported was set at 2. (b) Uniform manifold approximation and projection (UMAP) of cell subsets in the CITE-seq data. Numbers represent different Leiden clusters. (c) ROC curve on the testing dataset of all six classifiers. (d) Precision/recall curve on the testing dataset of all six classifiers. AP, average precision. (e), (f) Visualization of classifiers’ predictions in the (e) training data and (f) testing data embedded on the UMAP coordinates. The first subplot in each figure is visualization of the CD45RA true label in the CITE-seq data. The predicted CD45RA level of the testing data by Seurat 4 reference mapping is also showed in (f) (the rightmost subplot).

We found neural proliferation differentiation and control 1 (NPDC1), aquaporin 3 (AQP3), CCR6, IL7R, DPP4, HES6, CD28, and another 116 genes were qualified as differentially expressed (DE) in ${\text{CD}45\text{RA}}^{+}$ cells compared to ${\text{CD}45\text{RA}}^{-}$ cells under the standard that is often used in general scRNA-seq studies (|log_2_ FC| ≥ 2, *q*-value ≥ 0.05) (Fig. 1a and Supplementary Table S1). Among these DE genes, we found well-studied naive/memory signatures like CD28 and IL7R (Sallusto *et al.* 1999). Specifically, CD28 is a costimulatory receptor expressed on naive T cells, essential for T cell activation (Chen and Flies 2013). Interleukin 7 receptor (IL7R, alias CD127) is a receptor that binds to interleukin-7 (IL-7) and plays a crucial role in the survival, development, and homeostasis of naive T cells (Carrette and Surh 2012). Although previous literature described both naive and (stem) central memory T cells express CD28 and IL7R, the difference in their coefficients in the DE analysis indicates levels of these genes in ${\text{CD}45\text{RA}}^{+/-}$ cells differ.

Genes reported to be highly associated with T cells activation and differentiation, such as CD40LG, CTLA4, TBX21, and IL2RB, were also found in the DE genes. CD40LG encodes CD40 ligand, a protein activating and regulating the immune system, including T cells (Tang *et al.* 2021). CTLA4 translates into cytotoxic T-lymphocyte-associated protein 4, a protein that functions as an immune checkpoint and negatively regulates T cells activation (Schwartz 2003). TBX21, alias the gene of T-box 21, is a transcription factor involved in the differentiation of T cells (Seo *et al.* 2021). It was expected to see DE analysis report these genes, given they are considered highly indicative of the activation of T cells and partially in coordination with the expression of CD45RA.

We also found NPDC1 and AQP3 had the highest two log_2_ FC in ${\text{CD}45\text{RA}}^{-}$ group compared to ${\text{CD}45\text{RA}}^{+}$ group. NPDC1 is a protein-coding gene implicated in regulating cell proliferation, differentiation, and apoptosis in various cell types. Recently, it was reported to be a prognostic immune gene in a model that predicts the outcome of acute myeloid leukemia (Zhao *et al.* 2020). However, its specific role in T cell activation is not well-defined. AQP3 is a water channel protein that facilitates the transport of water and small solutes, such as glycerol, across cell membranes. AQP3 is expressed in various tissues, including the skin, kidneys, and gastrointestinal tract, and is involved in diverse physiological processes, such as water balance and skin hydration (Marlar *et al.* 2017). Its role in immune cells, specifically T cells, is not well-established, yet we did find its expression was highly overlapped with ${\text{CD}45\text{RA}}^{-}$, suggesting ${\text{CD}45\text{RA}}^{-}$ have a different metabolism compared to ${\text{CD}45\text{RA}}^{+}$ cells.

When determining the second source for feature selection, hnRNPLL’s expression was found to correlate with the transcription of HNRNPA2B1, a member of the hnRNP family, which plays a role in pre-mRNA processing, along with other 1600 genes that had a *P*-value > = .05 (Supplementary Table S2). Although HNRNPA2B1 has been reported to participate in alternative splicing (Zhao Peng *et al.* 2021), no direct evidence exists that it is associated with CD45 splicing specifically. Given its correlation with hnRNPLL, we suggest that it is possible that HNRNPA2B1 is also engaged in the CD45 splicing, inviting further investigation.

### 3.2 Performance of classifiers on the testing set and unseen data

The thresholds used for feature selection and the resultant features of all six classifiers (Table 1 and Supplementary Table S3) are recorded. The final values of the optimized hyperparameters (Table 2) are also reported. The accuracy (Table 3), precision (Table 4), and recall (Table 5) in training, testing, and the unseen datasets were used as metrics to evaluate the performance of classifiers. We found that all those simple classifiers achieved a reasonably good accuracy (>85%) in training, testing, and unseen, and the SVM with an RBF kernel outperformed the other four classifiers in terms of all three types of accuracy with a relatively parsimonious selection of features.

**Table 1.** Feature selection parameters.

Classifier	$t_{\text{DE–upper}}$	$t_{\text{DE–lower}}$	$P_{\text{hn}}$	$N_{\text{Features}}$
LR	1.131	−1.050	0.1	1349
SVM (linear)	1.793	−1.991	0.010	214
SVM (RBF)	1.689	−1.584	0.014	287
Stack	1.009	−2.534	0.073	1056

**Table 2.** The final values of hyperparameters.

Classifier	Hyperparameters
LR	$C_{\text{LR}}$ = 0.595
SVM (linear)	$C_{\text{SVM}}$ = 48.829
SVM (RBF)	$C_{\text{SVM}}$ = 54.343, *γ* = 0.069
Stack	$C_{\text{SVM}}$ = 1e-6, $C_{\text{LR}}$ = 0.054
MLP (linear SVM features)	$N_{\text{layers}}$ = 1e-4, $N_{\text{nodes}}$ = 0.1,
	*η* = 3, *ρ* = 105
MLP (RBF SVM features)	$N_{\text{layers}}$ = 2.262e-4, $N_{\text{nodes}}$ = 0.1,
	*η* = 1, *ρ* = 87

**Table 3.** Accuracy of predictions in three datasets.

Classifier	Training (%)	Testing (%)	Unseen (%)
LR	99.31	88.78	88.62
SVM (linear)	92.89	86.59	99.60
SVM (RBF)	99.45^a^	89.94^a^	99.96^a^
Stack	96.17	88.78	94.04
MLP (linear SVM features)	93.22	87.32	99.74
MLP (RBF SVM features)	95.48	89.21	97.63

a Marks the top performance model in each category.

**Table 4.** Precision of predictions in training and testing datasets.^a^

Classifier	Training (%)	Testing (%)
LR	99.39	88.33
SVM (linear)	92.38	88.56
SVM (RBF)	99.60^b^	90.20
Stack	97.04	90.87
MLP (linear SVM features)	94.70	92.31^b^
MLP (RBF SVM features)	92.91	88.76

a The precision and the recall of the unseen dataset are undefined as there are no positive instances to consider.

b Marks the top performance model in each category.

**Table 5.** Recall of predictions in training and testing datasets.^a^

Classifier	Training (%)	Testing (%)
LR	98.69	82.85
SVM (linear)	87.65	76.28
SVM (RBF)	98.90^b^	83.94^b^
Stack	92.27	79.93
MLP (linear SVM features)	86.14	74.45
MLP (RBF SVM features)	94.78	83.58

a The precision and the recall of the unseen dataset are undefined as there are no positive instances to consider.

b Marks the top performance model in each category.

Specifically, during the hyperparameter optimization, the LR classifier’s 5-fold cross-validation accuracy ranged from 62.77% to 90.42%, with an average of 86.79%. The linear kernel SVM’s 5-fold cross-validation accuracy ranged from 64.01% to 88.20%, with an average of 84.98%. The RBF kernel SVM’s 5-fold cross-validation accuracy ranged from 62.40% to 91.48%, with an average of 76.21%. The stacked classifier’s 5-fold cross-validation accuracy ranged from 84.95% to 91.00%, with an average of 88.60%. Lastly, the MLP using linear SVM features had a loss in the 5-fold cross-validation ranging from 0.2433 to 0.5151, with an average of 0.3355. The MLP using RBF SVM features had a loss in the 5-fold cross-validation ranging from 0.2221 to 0.6051, with an average of 0.3627. All classifiers consistently demonstrated strong performance as evidenced by their ROC curves and showed a balance between precision and recall, where the LR classifier and the SVM with an RBF kernel had the optimal averaged precision as shown in the precision–recall curve (Fig. 1d). Learning curves of classifiers also showed no sign of over-fitting (Supplementary Fig. S1).

**Figure 2.**
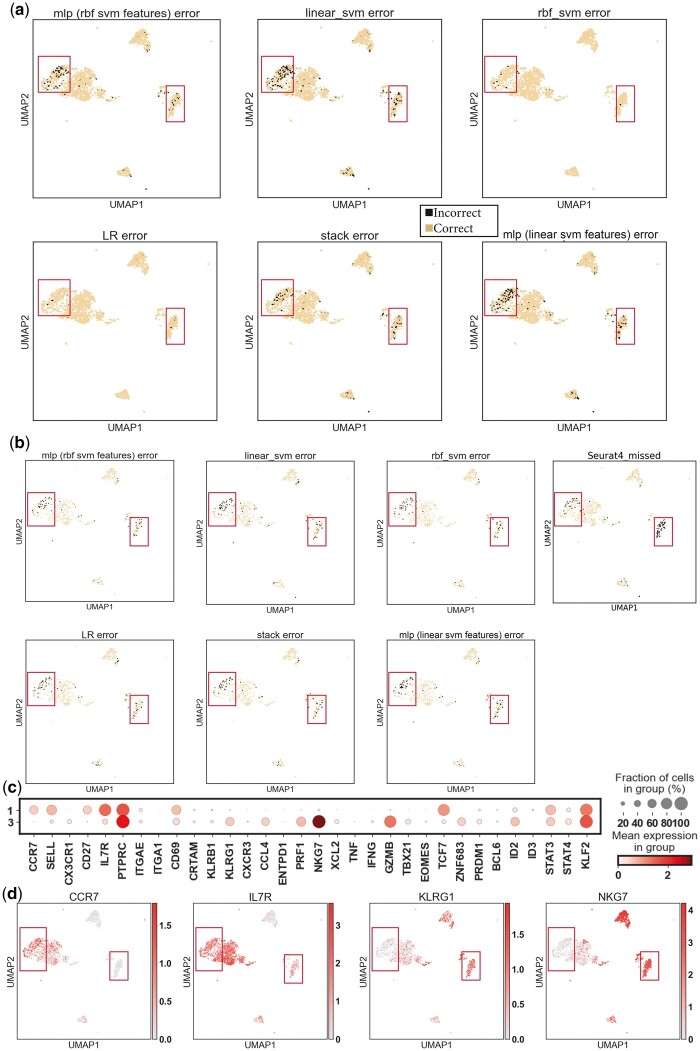
Misclassification and hard-to-classify clusters in the training/testing CITE-seq dataset. (a), (b) Visualization of classifiers’ wrong predictions (misclassification) in the (a) training data, and (b) testing data embedded on the .UMAP coordinates. In (b), the rightmost plot visualized the thresholded Seurat 4 CITE-seq reference mapping as a benchmark. (c) Expression (log-transformed corrected counts) of well-studied T marker genes in clusters 1 and 3 of the CITE-seq data. (d) Visualization of T subsets marker expression in CITE-seq data on UMAP.

The visualization of the classifier’s prediction and misclassification in training and testing sets showed that the misclassified cells mainly existed in two subsets of the CITE-seq data, Leiden clusters 1 and 3 (C1, C3), where CD45RA${}^{+}$ and CD45RA${}^{-}$ cells were mixed by general clustering (Figs 1b and 2a and b). The expression profile showed that the CCR7${}^{+}$SELL${}^{+}$IL7R${}^{+}$TCF7${}^{+}$ cluster 1 was likely to be naive T cells (CD45RA positive) or TCMs (CD45RA negative), and the CCR7${}^{-}$SELL${}^{-}$IL7R${}^{\text{low}}$KLRG1${}^{+}$NKG7${}^{+}$ cluster 3 had an obvious TEM (CD45RA negative)/TEMRA (CD45RA positive) similarity (Fig. 2c and d) (Traag *et al.* 2019). As discussed previously, these two pairs of cell types are difficult to classify in scRNA-seq analysis because of the overlap in the expression patterns and the absence of CD45RA, so it is reasonable that we observed misclassifications mainly came from these two clusters. Nevertheless, all six classifiers were still able to correctly label the majority of the hard-to-classify cells (Table 6). We also used Seurat 4 multimodal reference mapping on the validation set as our benchmark. To be more specific, Seurat 4 can map query scRNA-seq data to their 100k+ cells CITE-seq reference and predict the antibody-derived tag (ADT) of more than 200 proteins, including CD45RA. Applying Seurat 4 to the validation set following the instructions described in its vignette produced a predicted level of CD45RA expression in every cell, and it was shown that Seurat 4 assigned high levels of CD45RA to cells in C3, one of our hard-to-classify clusters, which is mainly CD45RA negative (Fig. 1f). Its performance serves as a testament to the complexity and unpredictability of the scRNA-seq data, hence the   ˜  80% accuracy achieved by our simple models may be considered a relative success in this specific instance.

**Table 6.** Percentage of misclassification in hard-to-classify clusters (training and testing).

	Training (%)		Testing (%)	
Classifiers	Naive/TCM (C1)	TEM/TEMRA (C3)	Naive/TCM (C1)	TEM/TEMRA (C3)
LR	1.21^a^	12.05	30.19^a^	19.32^a^
SVM (linear)	22.33	12.05	39.62	22.73
SVM (RBF)	2.19	0.65^a^	31.13	21.59
Stack	9.71	7.82	33.02	21.59
MLP (linear SVM features)	21.36	11.07	38.68	23.86
MLP (RBF SVM features)	14.56	6.84	32.08	22.73

a Marks the top performance model in each category.

**Figure 3.**
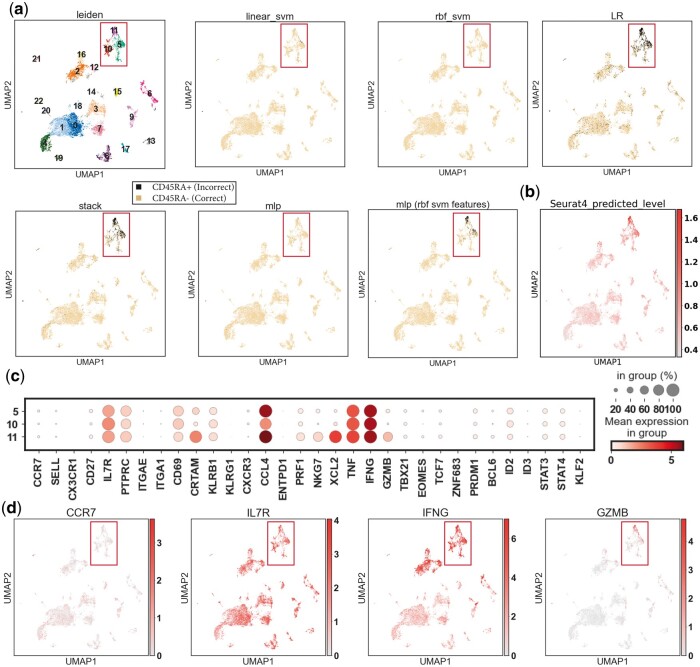
Misclassification and hard-to-classify clusters in the unseen dataset. (a) Visualization of classifiers’ predictions in the unseen data embedded in the UMAP coordinates. Given the unseen data were reported as all CD45RA−, which should not have any CD45RA+ cells, this plot also visualizes classifiers’ wrong predictions (misclassification) in the unseen data. The first subplot in each plot shows the Leiden clustering as a reference. (b) The predicted CD45RA antibody-derived tag (ADT) by Seurat 4 CITE-seq reference mapping. (c) Expression (log-transformed corrected counts) of well-studied T marker genes in clusters 1 and 3 of the CITE-seq data. (d) Visualization of T subsets marker expression in the unseen data on UMAP.

Applying the classifiers to a CD4${}^{+}$CD45RA${}^{-}$ unseen dataset allowed the evaluation of their performance when the input data are generated from a different experiment. Two SVMs and the MLP using linear SVM features achieved a high accuracy (>99%), and for the less accurate classifiers, LR and stacked models, the misclassifications were mainly from Leiden clusters 5, 10, and 11 (C5, C10, C11) (Fig. 3a and Table 3). Gene expression profiles of these three clusters showed they were likely to be resting (C5, C10; PRF1${}^{-}$GZMB${}^{-}$NKG7${}^{-}$) and activated (C11; PRF1${}^{+}$GZMB${}^{+}$NKG7${}^{+}$) CD4${}^{+}$ TEMs (expected to be CD45RA negative) given their CCR7${}^{-}$SELL${}^{-}$IL7R ${}^{+}$TNF${}^{+}$IFNG${}^{+}$ pattern (Fig. 3c and d). Indeed, CD4${}^{+}$ TEMs are also found to be able to re-express CD45RA (Tian *et al.* 2017), which makes these three clusters hard-to-classify if their CD45RA level is unknown. With that being said, the SVM with an RBF kernel perfectly labeled cells from these clusters based on their gene expression level (Table 7), and the other SVM with a linear kernel and the MLP using linear SVM features also performed well. We also queried the unseen data in Seurat 4 using their multimodal reference mapping, and it was shown that Seurat 4 assigned high levels of CD45RA to cells in C5 and C11, two of our hard-to-classify clusters (Fig. 3b). By comparison, our model’s success in correctly identifying the CD45RA negative cells in these clusters proved its usefulness despite of its simplicity. Based on the overall performance, we chose the SVM with an RBF kernel as the model implemented in our Python package scCD45RA.

**Table 7.** Percentage of misclassification in hard-to-classify clusters (unseen).

	Unseen		
Classifiers	TEM Rest 1 (C5)	TEM Rest 2 (C10)	TEM Act (C11)
LR	47.57	27.13	79.81
SVM (linear)	3.80	0.39	2.12
SVM (RBF)	0	0	0
Stack	36.52	13.44	70.58
MLP (linear SVM features)	0.82	0.39	2.50
MLP (RBF SVM features)	13.93	2.97	47.5

### 3.3 scCD45RA provides valuable insights into complex scRNA-seq data

**Figure 4.**
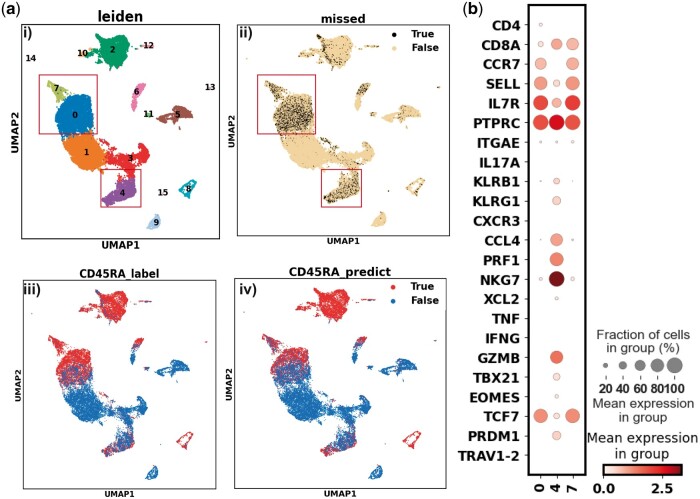
Misclassification and hard-to-classify clusters in the GSE164378 dataset. (a) (i) The Leiden clustering as a reference. (ii) Visualization of classifiers’ predictions in the GSE164378 data embedded in the UMAP coordinates. (iii) UMAP embeddings colored in the CD45RA true label of cells. (iv) UMAP embeddings colored in the predicted CD45RA label of cells. (b) Expression (log-transformed corrected counts) of well-studied T marker genes in clusters 0, 4, and 7 of the GSE164378 data. As can see in (a) (ii), most misclassified dots are in these three clusters.

Furthering our quest for a comprehensive validation, we turned to an additional independent dataset, GSE164378 (Hao *et al.* 2021), to provide a broader canvas for the robustness assessment of our model. It exhibited an accuracy of 82.31%, a commendable precision of 93.34%, and a recall of 70.73% on this dataset. A deep insight into the data’s complexity is evident from the Leiden UMAP overview (Fig. 4a, i). A closer inspection reveals that the most significant discrepancies in model predictions occurred in clusters 0, 4, and 7 (Fig. 4a, ii). When juxtaposing the true versus predicted CD45RA label distributions across the UMAP, we further underscore this observation (Fig. 4a, iii–iv). Yet, it’s crucial to contextualize these findings. Cluster 0, characterized as CD4^+^CCR7^+^SELL^+^IL7R^+^ and akin to a Naive/TCM phenotype, showed a 41.21% misclassification. Meanwhile, Cluster 7, with its CD8A^+^CCR7^+^SELL^+^IL7R^+^ profile suggesting a Naive/TCM-like orientation, faced a 27.42% misclassification (Fig. 4b). Lastly, Cluster 4, with its intricate TEM/TEMRA-like phenotype defined by CD8A^+^IL7R^mid^ CCL4^+^PRF1^+^NKG7^+^GZMB^+^ expression, experienced a 36.47% misclassification rate. While these clusters present challenges in delineating CD45RA expression, it’s pivotal to spotlight the broader perspective, which is that our RBF SVM model has demonstrated resilience and significant predictive capabilities across different datasets and can still provide useful insights in the general CD45RA level in the clusters. The in-depth analysis of the GSE164378 dataset doesn’t diminish the model’s value; instead, it enriches our understanding of its applicability and potential nuances in diverse experimental scenarios.

## 4 Discussion

This study explored several simple yet robust machine-learning classifiers to predict the CD45RA level of cells from its NGS scRNA-seq count matrix. We reported genes that were differentially expressed between ${\text{CD}45\text{RA}}^{+/-}$, and we suggested that the transcription of genes like NPDC1 and AQP3 could be potentially indicative of the CD45RA protein level in human T cells. We trained and optimized several classifiers and compared their performance in making an accurate prediction. Although all of them had acceptable accuracy, two nonlinear classifiers, SVM with RBF kernel and MLP with ReLu and Sigmoid activation functions (using linear SVM features), performed the best and almost perfectly predicted the CD45RA labels of unseen cells. Admittedly, the disparity in performance between the training and testing datasets for the LR and SVM (RBF) classifiers, as evident in Table 6, might raise initial concerns regarding overfitting. However, a deeper examination of our models’ learning curves showcases a notable convergence of training and validation accuracies as the training sample size grows. This behavior is a classic hallmark of models that resist the pitfalls of overfitting (Supplementary Fig. S1). Despite recognizing the observed performance gap between the datasets, it is crucial to highlight that LR and SVM (RBF) are not the terminal models incorporated into our Python package, ensuring the tool’s broader applicability and robust generalization.

The key advantage of this classifier is its ability to overcome the technical limitations associated with short-read sequencing, which has historically struggled with detecting CD45RA and isoform identification. Furthermore, unlike traditional multimodal reference mapping Seurat4 that takes minutes or even hours on large datasets (SatijaLab 2021), our method provides a fast, reference-free solution to the CD45RA label prediction. In five rounds of running, it takes about 47.52 s on average to process the GSE164378 dataset, which has 33 456 profiles on an average-performance [Intel(R) Core(TM) i5-8300H CPU @2.30 GHz, 16 Gb RAM] computer. Consequently, our classifier addresses an unmet need in single-cell transcriptomics and provides a simple, fast, yet valuable tool for immunologists. Recent advances in long-read sequencing technologies, such as Oxford Nanopore, have shown promise in addressing some of the limitations of short-read sequencing, especially in resolving complex isoforms and alternative splicing events (Jain *et al.* 2016). There is ongoing research and development to explore the use of long-read sequencing technologies, like Oxford Nanopore, for scRNA-seq (Philpott *et al.* 2021). However, despite these advances, long-read sequencing remains relatively expensive and less accessible for many researchers. Isoform prediction from raw sequencing reads has been an area of active research, with methods such as StringTie (Pertea *et al.* 2015) and Cufflinks (Trapnell *et al.* 2010) developed to address this challenge. While these methods have demonstrated success in predicting isoforms, they can be computationally intensive and may require specialized expertise.

The foundation of this study is the CITE-seq data, which simultaneously profiled the transcriptome and surface proteins at single-cell resolution (Stoeckius *et al.* 2017). As the technology matures and becomes more accessible, it is likely that the increased adoption of CITE-seq in the future will address the issue of CD45RA reporting as well as the identification of other difficult-to-report markers. However, since it is a relatively new technique that uses DNA-barcoded antibodies for protein detection, which increases the cost and complexity of the experiment, it has not yet become as popular as the conventional scRNA-seq. Therefore, it is anticipated that more conventional T cells scRNA-seq experiments will still be conducted. In this way, our model can provide insights into analyzing existing and incoming conventional scRNA-seq datasets easily and cost-effectively.

## 5 Conclusion

Our study presents a novel CD45RA${}^{+/-}$ binary classifier that addresses the challenges associated with short-read sequencing and provides an efficient solution for immunologists working with scRNA-seq data. Future work could focus on refining the classifier’s performance and extending its applicability to other cell markers or transcriptomic technologies. Moreover, integration with existing bioinformatics pipelines and tools could enhance its utility and enable researchers to uncover novel insights into the complex world of immune cell biology.

## Supplementary Material

vbad159_Supplementary_Data

## Data Availability

The data used for training and testing in this article are available in NCBI Gene Expression Omnibus at https://www.ncbi.nlm.nih.gov/geo/, and can be accessed with identifier GSE144434, GSE150132, GSE164378. The resultant package scCD45RA can be found at https://github.com/BrubakerLab/ScCD45RA and can be installed from the Python Package Index (PyPI) using the command “pip install sccd45ra.” The code used for processing datasets and training the models as well as all trained models can also be found in the repository under the branch “training.”
