# Supplementary material for: Enhanced annotation of CD45RA to distinguish T cell subsets in single-cell RNA-seq via machine learning
Source: Bioinform Adv. 2023 Nov 6;3(1):vbad159. doi: 10.1093/bioadv/vbad159 (PMC10676521; doi:10.1093/bioadv/vbad159)
Supplement: vbad159_Supplementary_Data [file vbad159_supplementary_data.zip › scCD45RA_Supplementary_Figure.pdf]

Enhanced Annotation of CD45RA to Distinguish T cell Subsets in Single Cell RNA-seq via Machine Learning
Author: Ran Ran, Douglas K. Brubaker
Supplementary Figure

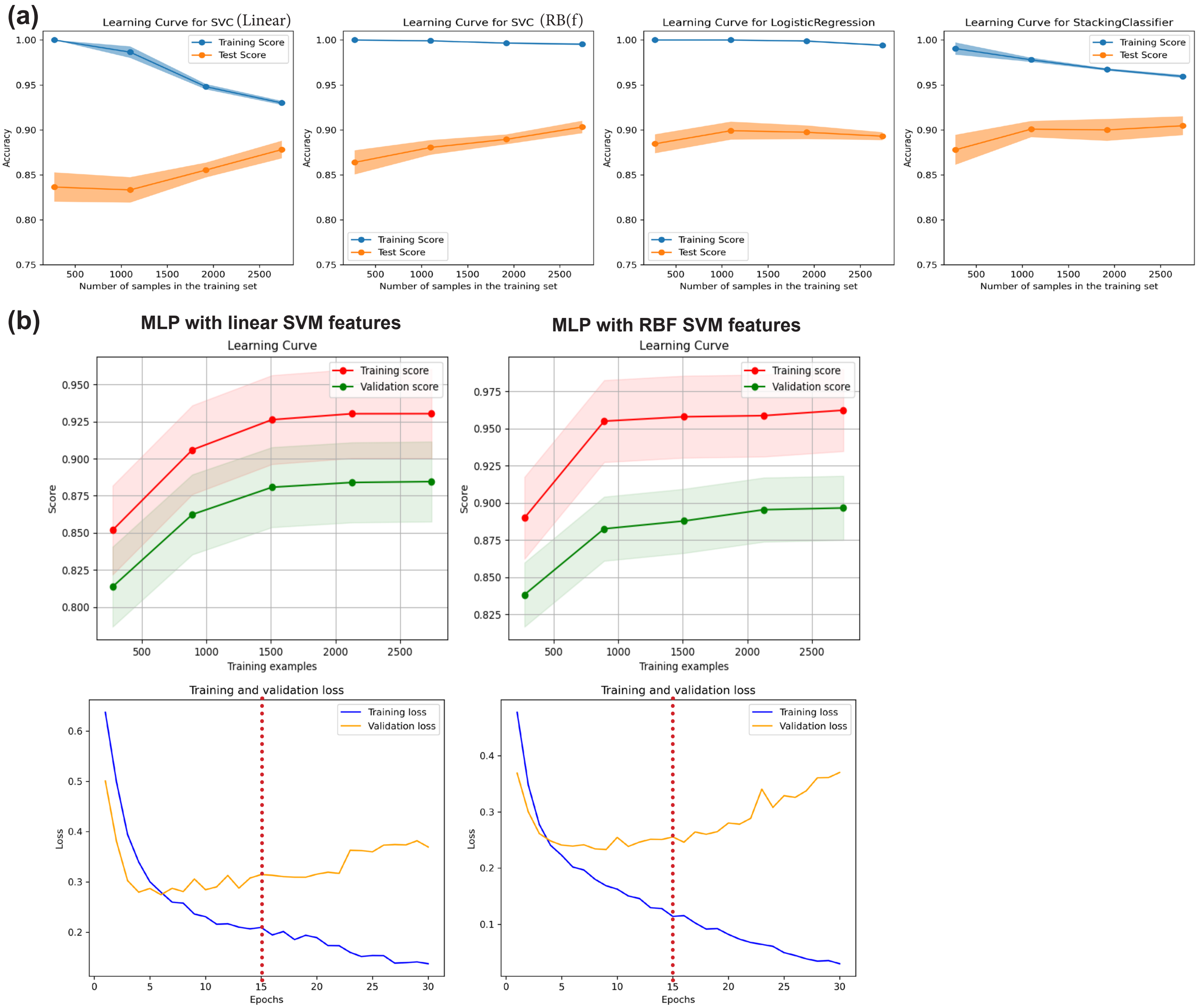

Figure S1. Learning curves of the trained models. **(a)**, Accuracies of linear SVM, RBF SVM, LR and stacked classifier on training (blue) and testing (orange) sets as the training size increases. **(b)** Top: Accuracies of MLPs using linear SVM features and RBF SVM features on training (red) and testing (green) sets as the training size increases. Bottom: Training (blue) and validation (orange) loss of different epochs. Red dashed line indicates the number of epochs used in the model (15).
